# Cancer and apoptosis: The apoptotic activity of plant and marine natural products and their potential as targeted cancer therapeutics

**DOI:** 10.3389/fphar.2022.842376

**Published:** 2022-08-10

**Authors:** Gul-e-Saba Chaudhry, Abdah Md Akim, Yeong Yik Sung, Tengku Muhammad Tengku Sifzizul

**Affiliations:** ^1^ Institute of Marine Biotechnology, Universiti Malaysia Terengganu, Kuala Terengganu, Malaysia; ^2^ Department of Biomedical Sciences, Faculty of Medicine and Health sciences, University of Putra Malaysia, Seri Kembangan, Malaysia

**Keywords:** apoptosis, marine drug, cancer, natural product, targeted therapeutic, apoptotic inducers

## Abstract

Cancer is a multifactorial, multi-stage disease, including complex cascades of signaling pathways—the cell growth governed by dysregulated and abrupt cell division. Due to the complexity and multi-regulatory cancer progression, cancer is still a challenging disease to treat and survive. The screening of extracts and fractions from plants and marine species might lead to the discovery of more effective compounds for cancer therapeutics. The isolated compounds and reformed analogs were known as future prospective contenders for anti-cancer chemotherapy. For example, Taxol, a potent mitotic inhibitor discovered from Taxus brevifolia, suppresses cell growth and arrest, induces apoptosis, and inhibits proliferation. Similarly, marine sponges show remarkable tumor chemo preventive and chemotherapeutic potential. However, there is limited research to date. Several plants and marine-derived anti-cancer compounds having the property to induce apoptosis have been approved for clinical trials. The anti-cancer activity kills the cell and slows the growth of cancer cells. Among cell death mechanisms, apoptosis induction is a more profound mechanism of cell death triggered by naturally isolated anti-cancer agents. Evading apoptosis is the major hurdle in killing cancer cells, a mechanism mainly regulated as intrinsic and extrinsic. However, it is possible to modify the apoptosis-resistant phenotype of the cell by altering many of these mechanisms. Various extracts and fractions successfully induce apoptosis, cell-cycle modulation, apoptosis, and anti-proliferative activity. Therefore, there is a pressing need to develop new anti-cancer drugs of natural origins to reduce the effects on normal cells. Here, we’ve emphasized the most critical elements: i) A better understanding of cancer progression and development and its origins, ii) Molecular strategies to inhibit the cell proliferation/Carcino-genesis, iii) Critical regulators of cancer cell proliferation and development, iv) Signaling Pathways in Apoptosis: Potential Targets for targeted therapeutics, v) Why Apoptosis induction is mandatory for effective chemotherapy, vi) Plants extracts/fractions as potential apoptotic inducers, vii) Marine extracts as Apoptotic inducers, viii) Marine isolated Targeted compounds as Apoptotic inducers (FDA Approved/treatment Phase). This study provides a potential therapeutic option for cancer, although more clinical studies are needed to verify its efficacy in cancer chemotherapy.

## 1 Cancer initiation and development: understanding cancer progression

**FIGURE 1:**
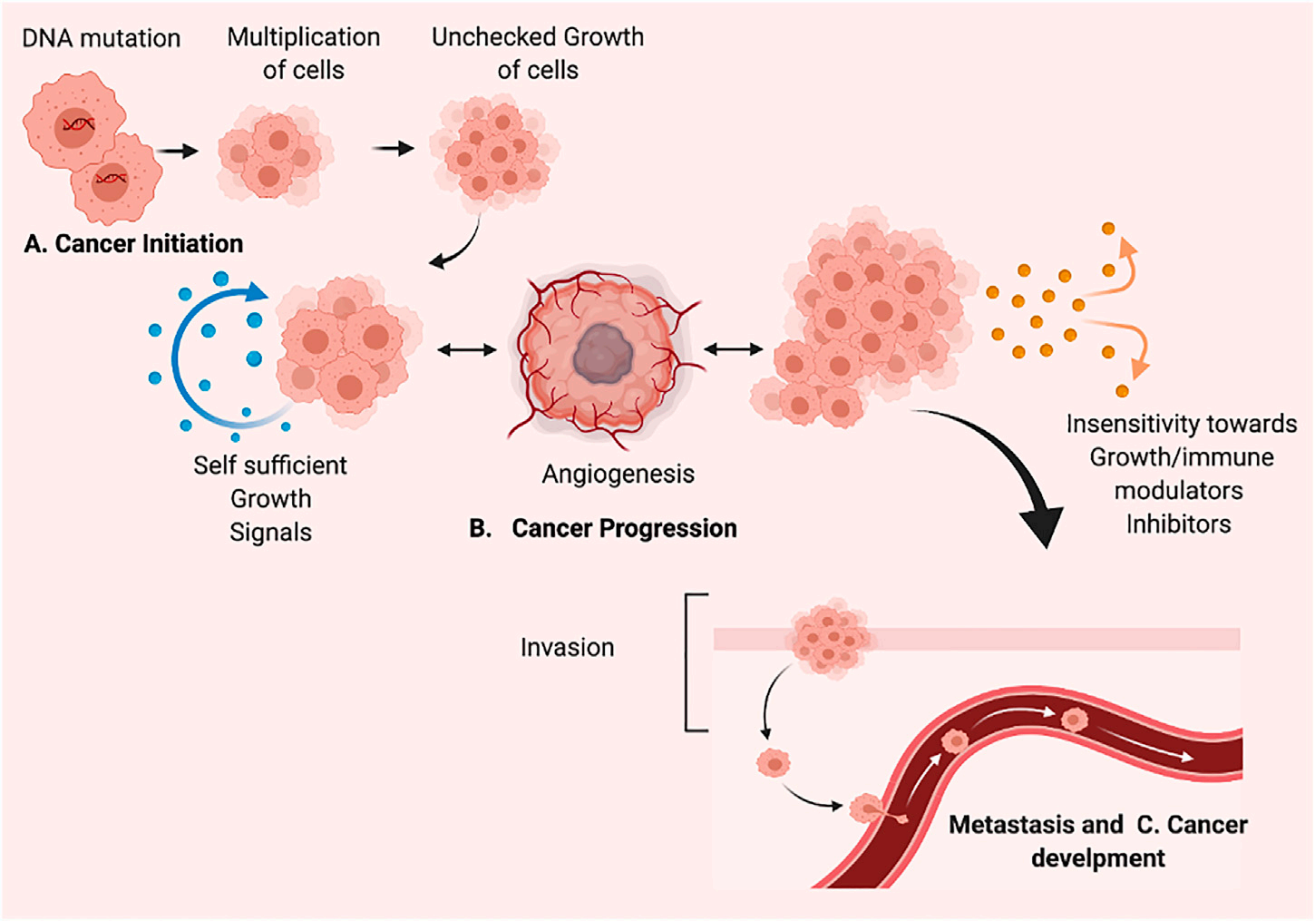
The process of cancer development/carcinogenesis, **(A)** Cancer initiation **(B)**. Cancer Progression **(C)**. Metastasis and Cancer development. Created with BioRender.com.

Cancer is the uncontrolled growth of specific cells possessed mutated genes or dys-regulatory cell division and growth. The word cancer means carcinoma, derived from a Greek word—cancer well-defined by the unregulated and uncontrolled multiplication of the cell. The specific alteration in genotype classified the normal cells as cancer cells responsible for uncontrol growth, re-location, and cancer metastasis (Sudhakar, 2009; Nithya et al., 2014; Fouad and Aanei, 2017). The cancer cell has a tremendous ability to divide and re-divide and metastatic abilities. The unchecked and over multiplication of cells leads to cancer tissue development and tends to move to various locations, resulting in metastasis development. In cancer, the different proteins involved in cell-cell attachment and regulation play a crucial role in regulating cancer cell movement. The matrix metalloproteases regulate the initiation and development of metastasis and enhancement of tumor spreading. The dysregulation in the expression of cell-cell attachment receptors fails cancer cell attachment. It encourages the ability to circulate in the bloodstream and re-locate to other locations inside the body—the abnormality in cell division, growth, and development results in cancer formation. The process of cancer development mainly comprises two fundamental alterations; i) genetical alternation and ii) Cellular alteration. The dys-regulatory genetic or cellular mechanism detected in cancer development. Genetically, the essential genes involved in the onset of cancer mainly comprised oncogenes and tumor suppression genes. The activation and deactivation of various “tumor suppressor genes” regulate the activation of cancer initiation and development. Furthermore, the variation in established genetic mechanisms involves genetic mutation, chromosomal abrasion or alteration, i.e., addition or deletion, and changes in cell cycling. The cellular alteration involves the dysregulated signaling pathways involved in the regulatory mechanism of dividing cells (Fearon and Vogelstein, 1990; Vogelstein and Kinzler, 2004). However, the hallmarks of cancer cells include: Selective proliferative advantage. i) Vascularization, immune modulation. ii) Metabolic rewiring. iii) Abetting microenvironment. iv) Tissue invasion/metastasis. v) Altered stress response. The cancer development process or carcinogenesis is mainly composed of diversified steps. Due to its rapidly dividing ability, the cell forms several cells or tumors and translocates to another site, leading to metastasis. Apart from general genetic and cellular alterations, epigenetic variations include; i) hypomethylation of oncogenes, ii) hypermethylation of tumor suppressor genes, iii) depletion of hydroxymethylation, iv) changes of histone acetylation, and v) methylation patterns and vi) miRNA expression level variations, are known to be associated with many cancers. Apoptosis is the primary mechanism that plays an essential role in balancing cells growth and cell division to prevent uncontrol cancer (Hassan et al., 2014). Carcinogenesis occurs due to damage, insult, or induction of alteration via various modes, including; physical, chemical, genetic, and biological. The cancer development and establishment process comprises cancer initiation, promotion, and progression (Figure 1). Various carcinogens act as initiators of cancer, which results in cancer formation along with promotor molecules. As in cancer initiation, most carcinogens cause irreversible damage to DNA. This damage causes the mutation in a particular genetic code that could be transferred to daughter cells and rapidly dividing cells with a mutated pattern. The cancer initiation trigger point could be an internal route or external via direct interaction with receptors on the cell surface or via various unspecified means. Thus, cancer initiation is based on irreversible genetic changes via the different genetic modes, including; i) simple mutations, small deletions, transitions, and transversions in DNA molecules. In the second stage of cancer development, there are no changes in the structure of DNA. The promotion of cancer is a reversible stage of advancement but rather in the expression of the genome mediated through promoter-receptor interactions. Interestingly, cancer progression depends on the protein involved in the migration of cancer. The extracellular environment (ECM) of cells is a pool of substances that could act like inducers that target the particular site and activate the cancer development or progression mechanisms. The last stage is characterized by cancer progression and migration, and karyotypic instability is considered an irreversible stage. However, the previous stage provides in-depth knowledge of molecular alteration, genetic changes in various tumor suppression genes, oncogenes, protooncogenes, and chromosomal aberrations (Faya et al., 2014). A better understanding of cancer etiology, initiation, and development of cancer has thoughtful consequences for targeting cancer biomarkers in developing therapeutics and considering agents/chemicals as carcinogens. In the cell death context, the fundamental process controlling the number of cells in tissue or organ development is apoptosis; however, in most cancers, the activation of oncogenes and dys-regulatory tumor suppressor genes occurs. The absence of apoptosis found in primary cancers makes the rapidly dividing cells unable to undergo the fundamental process of cell death.

## 2 Molecular strategies to inhibit the cell proliferation/carcinogenesis

**FIGURE 2:**
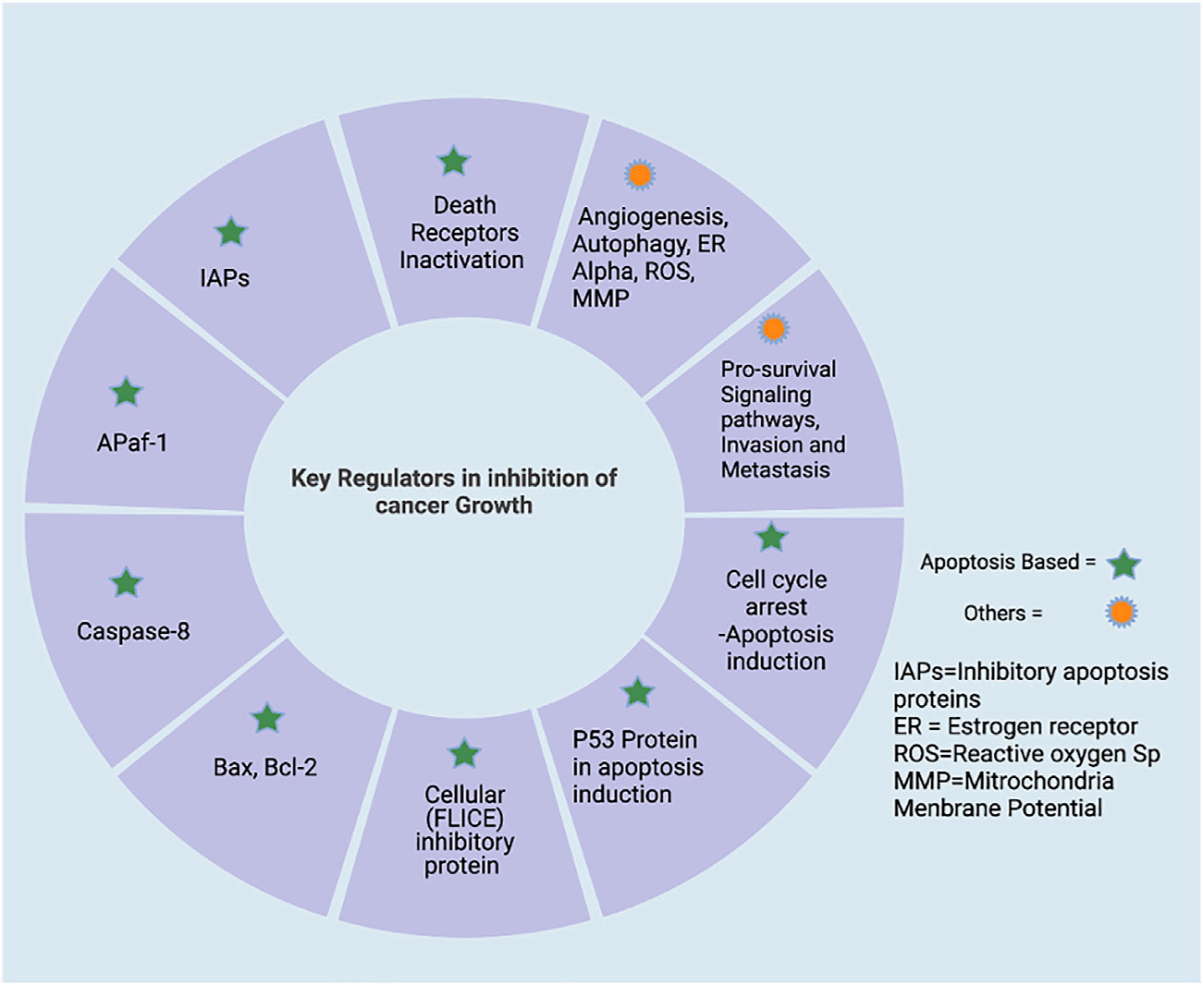
Targeting regulators in inhibition of cell progression. It is mainly divided into apoptosis regulators and others. Targeting Apoptosis includes i) up-regulation of pro-apoptotic (caspases protein, Bax, and pro-apoptotic member of Bcl-2 family) ii) down-regulation of anti-apoptotic proteins (IAPs). Also, by targeting the pro-survival signaling pathways, invasion and metastasis proteins dysregulated in cancer cells, created with BioRender.com.

Various potential players play an active role in inhibiting cell proliferation. Here, we highlighted apoptosis regulating proteins/elements and other than apoptosis-inducing factors as a targeted strategy to regulate the process of carcinogenesis or cell proliferation. The apoptosis regulating strategy begins with the cell cycle—the regulators involved in cell division, cell arrest, and induction of apoptosis. The various genetic regulators of cell proliferation hold a remarkable role in cancer cell division and growth (as shown in Figure 2). The cell cycle plays a critical function in cellular genomic integrity and cell progression (Hanahan and Weinberg, 2000). It is important to know that the cell cycle comprises various stages such as the first one, G1 (gap 1), as well as the second, G2 (gap 2), and the last one, M (mitosis). For genetic information to be passed down across generations, DNA synthesis and genome replication must occur during the S phase. The M-phase is characterized by the separation of genetic information, the development of sister chromatids, and cell division. The G1 phase is the transition from M to S, while the G2 phase is the transition from S to M. These breaks (G1 and G2) are necessary to ensure that each phase is completed before moving on to the next. (Jacobson et al., 1997; Fulda et al., 2010). Cell cycle checkpoints are frequently activated in response to replication stress and DNA damage. Cycle-dependent kinase activation and inactivation are critical for cell growth and cell cycle regulation. (Hanahan and Weinberg, 2000; Fulda et al., 2010). In normal cell division, there is tight regulation at each checkpoint. Any dysregulation in those checkpoints results in the rapid division of cells without the hindering. The rapid division increases cell growth. Similarly, up-regulating the various pro-apoptosis proteins/elements and down-regulating the anti-apoptosis proteins might be a positive regulators of apoptosis. The signaling pathway is described in Section 3. The p53 role in induction apoptosis could be enhanced by up-regulating the transcription of mRNA and translated product. The use of inhibitors to suppress the activation of inhibitors of apoptosis (IAPs) also aids in the successful establishment of apoptosis in cancer cells. The other strategy involves various regulator proteins involved in cell progression and growth, such as MMPs (matrix metalloproteinases), play a vital role in cancer progression. MMP inhibition or blockade is a critical target for reducing metastatic potential. In addition to MMPs, metastasis suppressor genes such as MKK4 (mitogen-activated protein kinase 4), BRMS1 (breast cancer metastasis suppressor 1), and NM23 (non-metastasis gene 23) play important roles in metastasis inhibition. (Kerr et al., 1972; Neuman et al., 2002). Conventional therapy options are exceedingly tough, particularly in patients with metastatic disease. Invasion, intravasation, and extravasation are part of the metastatic pathway. The spread of cancer cells to distant places via the circulatory system marks the invasion phase. On the other hand, extravasation necessitates cancer cells penetrating the endothelium and the basement membrane. Cancer cells can develop in a secondary focus in extravasation. (Neuman et al., 2002; Fulda et al., 2010). Additionally, regulation of uPA and uPAR expression and TIMP expression are critical for metastasis prevention. (Kanduc et al., 2002). A complex net of signaling molecule cascades largely transduced several “pro-survival signaling pathways” that determined the fate of a cancer cell. Several cytokines and growth factors trigger pro-survival signaling cascades such as IP3K-PKB/Akt and MAPK. Nuclear factor-B (NFB) is a protein that plays a crucial function in controlling inflammation and immunological responses (Hakem and Harrington, 2005). The importance of blocking these pro-survival signaling pathways in various cancers has been thoroughly researched. Angiogenesis, autophagy, reduction of ER expression, intracellular ROS decrease production, and activation of “mitochondrial membrane potential” are among the signaling pathways that cause cytotoxicity in cancer cells.

## 3 Signaling pathways in apoptosis: Potential strategy for targeted therapeutics

Apoptosis is an essential physiological process of cell death that is meant to occur without the discharge of intracellular contents, and subsequent no activation of an inflammatory response as apoptosis is a crucial process in embryonic development, regulation of the immune system, and the response to DNA damage. However, dysregulation of apoptosis results in significant consequences in carcinogenesis. The imbalance between cell proliferation and cell death is considered the malignant tumor’s hallmark (Giorgi et al., 2008). Therefore, maintaining cellular homeostasis between proliferation and cell death is essential for normal physiological processes.

**FIGURE 3:**
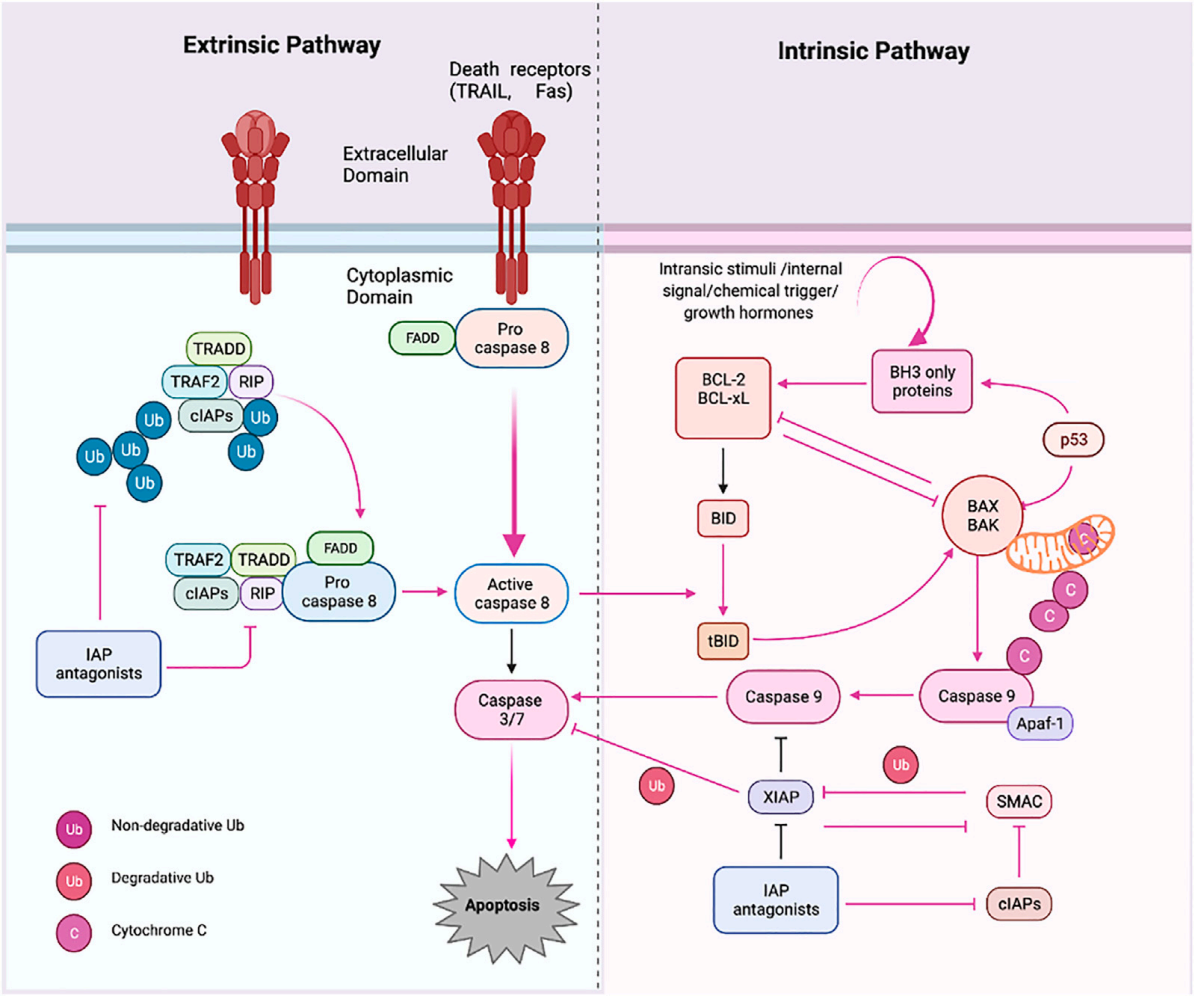
Apoptosis pathways. Apoptosis has two primary routes known as the i) extrinsic and ii) intrinsic pathway. First, external stimuli or ligand molecules activate the transduction, including death receptors (SDRs), leading to caspase 3/7 activation via/or activated caspase 8. Intrinsic pathway; via insertion of proapoptotic molecules BAX (protein) into the mitochondrial membrane results in the generation of cytochrome c, forming an apoptosome, which further triggers the apoptotic cascades beginning with proapoptotic activation caspase 9 and or then caspase 3. Created with BioRender.com.

The signaling molecules of the apoptotic pathway played an essential role in the regulation of the apoptosis process. Therefore, these molecular proteins might be considered potential apoptotic biomarkers that can be targeted in advanced cancer treatment and therapeutics. Due to the development of potential biomarkers in cancer, targeted therapy research has emerged as an effective tool for cancer treatment compared to the conventional method. The standard chemotherapies act on rapidly dividing normal and cancerous cells, whereas targeted therapy plays an influential role as they act upon specific molecular targets related to specific cancer. The targeted apoptotic biomarkers in cancer are in clinical trials. However, the targeted therapies are designed so that they interact with their target, whereas many standard chemotherapies were identified because they kill cells. Furthermore, targeted therapies are often cytostatic as they block tumor cell proliferation, whereas standard chemotherapy agents are cytotoxic and kill tumor cells). Therefore, the apoptotic biomarkers and chemotherapy could combine targeted drug therapy to get the maximized results. Apoptosis has been extensively investigated over the last two decades. It is now a critical process of controlled death activated in response to cell injury and during normal “development and morphogenesis.” For instance, the apoptotic death of nearly half of newly produced peripheral neurotransmitters throughout formation forms the nervous system of vertebrates to regulate their quantity, so it matches the requirements of their target organs in the peripheral (Alberts et al., 2002). Induction of apoptosis is essential for complex organisms’ embryonic development and differentiation equilibrium. It occurs in a controlled manner, with characteristic morphologic markers such as “cell shrinkage, “chromatin condensation,” and “blabbing of the cytoplasmic membrane.” Apoptosis dysregulation has indeed been linked to several pathologies, notably tumor growth and the formation of cancerous cells’ chemoresistance (Gorski and Marra, 2002). Among mammals, apoptosis is triggered by two well-known pathways (Figure 3). Extrinsic signals, such as TNF “tumor necrosis factor,” Fas (CD95/APO1), and TRAIL (TNF-related apoptosis-inducing ligand receptors), might induce apoptosis. In contrast, internal stimuli, including mitochondrial transduction, also can trigger apoptosis. For example, the activation of cysteine aspartyl proteases (caspases) causes permeabilization of mitochondrial membranes, chromatin condensation, and DNA fragmentation, resulting in cell death (Zornig et al., 2001).

The intrinsic route is triggered by physical and chemical inputs, including hypoxic conditions, growth factor shortages, cell detachment, and stress signals. The initiator caspases activation activates effector caspases; these are “aspartate-specific proteases” regulated post-translationally. Caspase inside as inactive proenzyme consists of a prodomain (a small and large subunit), which must be oligomerized and cleaved to activate. On the other hand, Caspases-independent apoptosis has been documented (LeBlanc, 2003; Bredesen et al., 2006). The death ligand binds the intracellular death domain of death receptors to begin the extrinsic pathway (Duckett et al., 1998; Venderova and Park, 2012). Many apoptotic signals are relayed to cell death machinery by p53, which interacts with other proteins like TNF, Fas, and TRAIL receptors, which are particular physiologic mediators of the extrinsic signaling route of apoptosis. Mitochondria have a role in several vital processes, including the caspase activators (cytochrome C), alterations in electron transport, mitochondrial membrane potential regulation, and the involvement of both pro-and antiapoptotic Bcl-2 family proteins (Bibel and Barde, 2000; Johnstone et al., 2002).

The proteins involved in the intrinsic route are “SMAC/DIABLO, Caspase-9, Bcl-2, Bcl-w, and Nox (Green, 2005). The intrinsic mechanism involves “Bax/Bak membrane insertion and cytochrome c release from the mitochondrial intermembrane gap into the cytosol (Anichini et al., 2006). The apoptosome is a multi-protein complex consisting of a “seven-spoke ring-shaped complex” that activates caspase 9 and then the caspase-3 signaling cascade, which leads to cell destruction and apoptosis (Hengartner, 2000). According to experimental findings, caspases have an overwhelming role in apoptosis (Ashkenazi and Dixit, 1998). There is a hopeful future for cancer therapy techniques that induce apoptosis in cancer cells by disrupting mitochondrial biogenesis, outflowing matrix calcium glutathione, or releasing membrane proteins due to mitochondrial failure. Anti-apoptotic proteins Bcl-2 and Bcl-xL block the cytochrome c release (Block et al., 1992). The development of a death-inducing signaling complex DISC which is made up of the adaptor molecule Fas-associated death domain (DD), procaspase-8, procaspase-10, and the cellular FLICE inhibitory proteins, is caused by the activation of SDRs by specific death ligands (DLs) (Heinrich et al., 1998). This Caspase 8 is activated in a real way that the “prodomain of caspase 8″ remains attached to the DISC. In contrast, the “active caspase 8 dissociates from the DISC” to initiate the cascade of caspase activation that occurs during the “execution phase of apoptosis (Kreuger et al., 2012). Traditionally, “caspases have been divided into i) initiator, ii) effector, and iii) executioner” types based on where they are located in an apoptotic signaling chain. At the same point, both extrinsic and intrinsic paths converge (execution phase). The execution phase is the last step in the apoptotic process (Kim, 2005; Elmore, 2007). Certain caspases have lengthy pro-domains that contain specific motif sequences, such as the death effector domain (DED) and caspase recruitment domains (CARD), which enable them to interact with other proteins and connect to signaling pathways. Caspases-1, caspase-2, caspase-4, caspase-5, caspase-9, caspase-11, and caspase-12 are involved in DED, whereas caspase-1, caspase-2, caspase-4, caspase-5, caspase-9, caspase-11, and caspase-12 are involved in CARD (Yuan and Akey, 2013). Caspases are critical apoptosis initiators and executors, and their function is strongly linked to their structure, which has distinct substrate preferences. The “Caspase-8 and 9 are initiator caspases, while Caspase-3, Caspase-6, Caspase-7, Caspase-10, CAD, and PARP” are effector or executioner caspases (Stergiou and Hengartner, 2004; Ghobrial et al., 2005). Caspase initiators are triggered by autocleavage, activating executioner caspases, which later proteolyze certain substrates, resulting in apoptosis. They have extended pro-domains that link big adaptor molecules, allowing them to multimerize and activate other caspases. However, effector caspases, on the other hand, have “short pro-domains” that execute apoptosis when activated by initiator caspases. As a result of the cytoplasmic endonuclease activated by executor caspases, chromatin condenses, cytoplasmic blebs develop, and apoptotic bodies are formed. Numerous target proteins are cleaved by caspases to regulate apoptotic cell death (Green, 2005). Caspases 3 and 4 are the essential executioner caspases, and an initiator caspase can activate them. Caspases-3 specifically activates endonuclease CAD, resulting in chromosomal DNA degradation and chromatin condensation inside nuclei. The cytoskeletal rearrangement and generation of cytoplasmic blebs and apoptotic bodies are both dependent on execution caspases (Medema et al., 1997). Execution caspases are activated first, followed by cytoplasmic endonuclease activation. Interestingly, nuclear material is degraded by cytoplasmic endonuclease, and proteases then degrade nuclear and cytoskeletal proteins.

## 4 Why apoptosis induction is mandatory for effective chemotherapy

An anti-cancer treatment mainly kills cancer cells and may or may not cause side effects to healthy cells. Currently accepted cancer therapy methods include a combination of medications, surgery, radiation, or a combination of all of these. Chemotherapeutic drugs can relieve symptoms, extend life, and even cure cancer in some cases. One of the best cancer drugs has a low risk of harming healthy cells in treating patients. Apoptosis substantially impacts both the lifespan of healthy cells and the prognosis of malignant cells. As a result, regulating default apoptosis could benefit cancer treatment and prevention. The aberrant response of malignant cells to apoptosis induction is caused by excessive cellular proliferation, which can also be described as overexpression of inhibitors or IAP family members. The Cell cycle-regulating genes are inactivated in cancer cells, and Bcl-2) adjusts their expression in tumors. Different malignancies, inflammatory illnesses, viral infections, and autoimmune diseases are caused by inhibiting or suppressing apoptosis.

Interestingly, apoptotic induction has emerged as a novel target for novel mechanism-based drug discovery (Ghavami et al., 2009; Sankari et al., 2012). Previously, it was thought that an increase in cellular proliferation was only linked to the rise in the number of cells accumulating in the body; however, it has now been discovered that a decrease in cell death is also responsible for cell proliferation. As a result, it is crucial to screen apoptotic inducers from natural products, either as crude extracts (plants or animal/marine organisms) or as separated components. Various studies have found evidence that compounds derived from natural sources effectively treat and prevent cancer. The isolated secondary metabolites from multiple natural sources, and mechanistic study (*in-vitro*), give better molecular fundamental knowledge of the future therapeutic agent. Various plants extracts, fractions, synthetic palladium, and other complexes are used to screen cytotoxicity and mode of cell death, which reduces the cost of isolation of phytochemicals and synthetic compounds and gives insight into a potential therapeutic agent. The ability to trigger apoptosis may serve as a unifying principle for many distinct types of chemo preventive drugs, each with a unique mode of action. Also, insight into the mechanisms of action of these chemicals could lead to new cancer-preventive and treatment strategies. Synthesis or modification of previously recognized medications in cancer treatment is still a significant research focus. Despite massive amounts of synthetic effort, the final products have improved slightly over a few prototypes. Thus, there is a constant demand for new chemotherapeutic agent prototypes–templates to develop more effective chemotherapy. Natural products (NPs) serve as models for new prototypes in the search for drug discovery. Apart from chemotherapy treatment, epidemiological studies suggest that eating lots of fruits and vegetables, rich in phytochemicals and other nutrients, may lower one’s risk of getting cancer (Jan and Chaudhry, 2019). In addition, it has been shown that natural product-derived tumor inhibitory chemicals have revealed a remarkable array of novel structural forms.

## 5 Plants extracts/fractions as potential regulator of pro-apoptotic and anti-apoptotic agents

Apoptosis is a cell death process that is conserved and well-controlled. It is essential to complex organisms’ development and homeostasis. Apoptosis inducers work very well in causing cytotoxicity in cancer cells. Due to their selective targeting of cancer cells, dys-regulatory apoptosis machinery only targets normal cells compared to cytotoxic agents. Therefore, they can work along with potential chemotherapy and reduce the risk of re-occurrence via killing the cell in a natural suicidal way. One of the essential factors in combination medication therapy is awareness of diverse phytochemicals and their synergistic approach. By indicating the presence of a potential agent, the extracts/fractions screening lowers the cost of phytochemical separation. Apoptosis’s external and intrinsic triggers include; radiation, growth factor withdrawal, ligands, tumor suppressor protein (p53), and oxidative stress (Hengartner, 2000; Hu et al., 2013). Additional pathological activities associated with the physiologic apoptosis pathway include cancer, inflammatory, and neurological diseases (Cairrao and Domingos, 2010). As shown in a substantial body of research, many cancerous tumors have a decreased capacity to die by apoptosis in reaction to physiological stimuli (Los et al., 1995). Therefore, it is critical to broadening the chemical repertory of agents that might induce or sensitize resistant cells to apoptosis to combat cancer. Furthermore, employing natural compounds as anti-carcinogenic or cytotoxicity-inducing compounds may be promising cancer prevention and therapy strategy. An *in vitro* investigation of secondary metabolites from various natural sources provides a better understanding of the molecular fundamentals of the therapeutic drug in the future (Israels and Israels, 2000).

### 5.1 Alkaloids based apoptosis induction

Alkaloids are chemical compounds found in nature that are predominantly made up of basic nitrogen atoms, neutral groups, and mildly acidic (Pucci et al., 2000; Khazaei et al., 2017a). Alkaloid Solamargine, located in the Chinese Plant Solanum incanum, has been demonstrated to trigger apoptosis in cancer cells (Hep-3B) and standard (“skin fibroblast cells”). Also, the level of TNF receptor I significantly elevated after solamargine treatment (Martin et al., 2000). Necrophilia guyanensis and Genipa Americana extract fractionation contain alkaloid cryptolepine, which appeared as the predominant active component. The cryptolepine compounds demonstrated substantial cytotoxic effects in human cells via the underlying mechanisms of apoptosis (Decock et al., 2011). Camptothecin (CPT), an inhibitor of topoisomerase I, causes triggered Oxygen radicals which cause DNA fragmentation and eventually induction and completion of apoptosis. (Cox et al., 1999).

### 5.2 Terpenes based apoptosis induction

The monoterpenes D-limonene and perillyl alcohol were found to have anti-carcinogenic properties in various cancer cell lines by exhibiting apoptosis. For example, perillyl alcohol was very effective in causing cell death of breast cancer cells (Klener et al., 2006). Similarly, Limonene inhibits stomach carcinogenesis caused by sodium chloride by enhancing apoptosis (Reddy et al., 1997). Taxol is a cytotoxic diterpene discovered in *Taxus brevifolia* that suppresses cell growth and proliferation. The mechanism of action is triggered via stabilizing power of Taxol during the spindle fiber formation in the cell division stage (mitosis). The polymerization of the Microtubule and improved stimulation of the formation of microtubule (MT) bundle impeded entry into the S phase, limiting cell division and apoptosis and also triggering necrosis at higher concentrations of Taxol (Fisher David, 1994). However, Paclitaxel-induced cell death also occurs via a signaling pathway not dependent on G2/M arrest (Pistritto et al., 2016).

### 5.3 Polyphenols based apoptotic inducers

Polyphenols obtained from Plant are abundant in the human diet and are deemed healthy at the recommended dose (one G/day) (Ren and Tang, 1999; Chaudhry et al., 2021a). A range of mutated cell lines (Schultze et al., 1991) show that gallic acid predominantly induces cell death. Similarly, in HL-60 cells, caffeine and tannic acids induce DNA breakage (Hajto et al., 1989). CAPE, the main ingredient in traditional medicinal propolis, has been shown to have particular cytotoxic activity against mutated cloned rat embryo fibroblast (CREF) cells by apoptotic cell death (Liu et al., 2000). CAPE, the main ingredient in traditional medicinal propolis, has been shown to have particular cytotoxic activity against mutated cloned rat embryo fibroblast (CREF) cells by apoptotic cell death (Liu et al., 2000). It has been established and demonstrated that curcumin, the primary color in turmeric, is a phenolic compound that causes apoptosis—a beneficial chemotherapeutic addition since it does not affect normal cells. The activation of apoptosis was boosted by curcumin’s ability to block cyclooxygenase (COX) products (Buhagiar et al., 1998; Bussing et al., 1999). Moreover, chilli pepper and hot red pepper contain capsaicin, triggering HL-60 cells to undergo apoptosis (Reed and Green, 2002).

### 5.4 Xanthones based apoptotic inducers

Xanthones are found in the naturally occurring substances in both plants and microbes. This new species from marine fungi contains anti-cancer xanthone compounds, which have been noticed recently. This is established that toxic compounds and anti-carcinogenic medication breakdown and elimination affect certain proteins. Xanthone compounds with anti-carcinogenic characteristics may be found in aquatic species and microalgae. In mangostana’s pericarp -mangostin, the xanthone induced apoptosis via up-regulation of caspase-3 (Thompson, 1995; Ashkenazi and Dixit, 1998).

### 5.5 Plants extracts regulate pro-apoptotic and anti-apoptosis proteins

The Table 1 shows the list of potential plant extracts as apoptotic inducers. Pepino, the extract was essential for the onset of DNA fragmentation and PARP cleavage (Ng and Bonavida, 2002). Similarly, *Viscum album* L. (*Loranthaceae*) extract in human lymphocytes causes cytotoxicity, more probably via apoptosis (Gorczyca et al., 1993; McNaught and Andrew, 1997). *Salvia miltiorrhiza* is often used to cure liver problems throughout Asia for centuries. Investigators found that sage *salvia miltiorrhiza* extracts showed significant cytotoxic activity and could have reduced the human hepatoma (HepG2) cell line (Manske Richard Helmuth, 1965). Apoptosis was shown to drive the cytotoxic activity of *Conifer Tetraclinis articulate* (essential oil) on several human cancers, including blood lymphocytes (Shu-Hui et al., 1996). Bussing et al. employed a seed extract of these and discovered that it produced oxygen radical mediators and induction of apoptosis. The “tumor necrosis factor” (alpha) and “interleukin-6" (IL-6) constitute pro-inflammatory factors, whereas “interleukin-5" (IL-5) and “interferon-gamma” (IFN-g) are T-cell associated cytokines (Shu-Wei, 1999). *Genistein* (a hydroxyisoflavone) was found to induce cell death in human promyelocytic (HL-60) leukemic cells according to flow cytometric analysis (Hiraoka et al., 1998). Vitex rotundifolia (leave) fraction remarkably induces apoptosis in MCF-7 & T47D via extrinsic and intrinsic pathways (Ariazi et al., 1999; Yano et al., 1999; Yeung et al., 1999). Squamous cell carcinoma was inhibited by an ethanolic extract of *Azadirachta indica* (neem) leaves in a hamster cancer model. The presence of extract increased the pro-apoptotic proteins (caspase-3 and -8, BIM, and Bcl-2), indicating that both internal and external mechanisms were significantly impacted. The Bax and Bcl-2 are up-regulated in breast cancer cell lines (MCF-7 and MDA-MB-231) by Phaseolus *vulgaris* (*Fabaceae*) extract treatment in cells, respectively (Bcl-2, Bcl-xL) (Weimin, 1999). In addition, a methanolic extract of Fragaria ananassa (Strawberry) increased Bax, Bid, and p73 expression in T-47D cells while decreasing Bcl-xL expression (King-Thom, 1997). According to apoptosis-inducing meisoindigo, the receptors on the cell surface and mitochondria are involved, according to the study. The colorectal cancer cell lines, methyl ferulate, a Tamarix aucheriana plant product, enhanced caspase-2, -3, -6, -7, -8, and -9 expression while decreasing the anti-apoptotic proteins Bcl2 and FLIP (Meyer et al., 2005). *Inula racemosa* (also known as pushkarmool) roots induced apoptosis and necrosis in human leukaemia cell line HL-60 when treated with an ethanolic extract of *Inula racemosa* roots. While cytochrome C (release) and Bax (translocation) dramatically reduced, this substance boosted the activity of Caspase-9, -3, and -8 (Makoto et al., 1994). The activation of intrinsic caspase-6, -8, and-9 by *Oenocarpus bacaba* aqueous extract promoted apoptosis in MCF-7 cells (Sakagami et al., 1994). *Solanum lyratum* (known as nightshades) chloroform extract is triggered by the extrinsic and intrinsic apoptotic mechanism in HSC-3, CAL-27 & SAS cancer cells. Among the cell lines studied, the extract decreased anti-apoptotic proteins (Bcl-2 and Bcl-xl) while elevating pro-apoptotic (Bax and Caspases) (Su et al., 1994). The cardiac glycoside (Ouabain) significantly decreased ROS and MMP levels while increasing Ca^2+^, activation of pro-apoptotic members, and reduced expression of Bcl-2 levels (Rao Chinthalapally et al., 1995). The cardiac glycoside (Ouabain) significantly decreased ROS and MMP levels while increasing Ca^2+^, activation of “pro-apoptotic” members, and reduced expression of Bcl-2 levels (Rao Chinthalapally et al., 1995). Cell cycle arrest and apoptosis were caused by “Rosamultic acid,” a triterpenoid, which cleaves PARP and activates caspase-3, -8, and -9 in SGC-7901 cells (Rao et al., 1995). A flavonoid produced from plants, Acacetin triggers an apoptotic cascade in stomach cancer cells (AGS cells) and kills them. “When ROS are produced, MMP collapses and “Bax and p53″ are increased “Bcl-2 is decreased”. Extrinsic activation of “caspase-8 and Bid proteins” is facilitated by an increase in the expressions of Fas and FasL (Wolvetang Ernst et al., 1996). Caspase-8 and Caspase-7 were activated in breast cancer (MCF-7) cells after exposure to extracts (methanol and & butanol) of “*Oldenlandia diffusa”* (Willd) Roxb. As a result, Bax expression was increased, and Bcl-2 levels were decreased (Matsumoto et al., 2003). Cell death was prevented in MCF-7 cells by *Cucurbita ficifolia* (chloroform) extract. There was an increase in the expression of FADD, BAK, BAX, and caspase-8, -9, and -3 (Nakagawa et al., 2007). Lycorine, an alkaloid molecule (from the *Amaryllidaceae* family), has been shown to enhance the “Bax/Bcl-2″ protein ratio and the activity of caspase-(8, -9, and -3) enzymes in cells compared to untreated cells (Chaudhry et al., 2019a). A mango peel ethanolic extract (*Mangifera indica*) has been shown to cause the death of HeLa cells. As a result of activating pro-apoptotic proteins (caspase-3, 7, 8 & 9) and the suppression of Bcl-2 (Chaudhry et al., 2019b). Celastrol, a pentacyclic triterpenoid, induces apoptosis via acting on receptor protein (death receptor) and internal pathways. Celastrol stimulated the signal transduction in mitochondria and increased Bax while decreasing Bcl-2 protein levels in the intrinsic route. To increase the expression of FAS and FASL, it induced FASL expression in the extrinsic pathway. Because celastrol activated caspases 9, 8, 3, and PARP, these enzymes began to break down proteins. Using a mouse model of cancer, Celastrol prevents the development of human glioblastoma xenografts by suppressing angiogenesis. It also induces apoptosis in osteosarcoma cells and increases the activities of pro-apoptotic caspase-3, -8, and 9 (Verdine, 1996; Chaudhry et al., 2020a). An ethanolic extract of *Brucea javanica* fruits was discovered to have apoptotic properties when tested on colon cancer (HT29) cells. The drug’s mode of action involves both receptor- and mitochondrial-mediated processes. The extract also increased TNF2 and DR6 in the extrinsic route. Caspase-8 and TRAIL-4 were also increased. Caspase-9 was also activated, which led to an increase in Bax, Bad, and cytochrome-c while decreasing Bcl-2 (Subapriya et al., 2005). Experiments on human cancer cell lines have shown that plant extracts can activate intrinsic and extrinsic pathways to cause cell death. They were also beneficial *in vivo*, killing cancer cells and delaying cancer progression. Because of this, even though these plant extracts have long been used in traditional cancer treatments, western science and medicine need proof of their anti-cancer properties using pure plant compounds isolated from plants. The intrinsic and external mechanisms in adult tumor cells can be affected by an N, N-dimethyl plant sphinganine (DMPS) derivative known as phytosphingosine (PS) (HL-60 cells). -Caspase-8 activation is a major element at the beginning of the intrinsic pathway, followed by the cytochrome C release and activation of pro-apoptotic proteins (caspase-9 and caspase-3, and inhibition of Bcl-2 (anti-apoptotic members of the family (Abadio Finco et al., 2016). Anthocyanins, a kind of flavonoid, are well-known for their medicinal qualities. Anthocyanin components produced from *Vitis coignetiae* destroy human leukaemia (U937) cells by apoptosis induction in these cells. Bax levels rise when Bid and caspases 3, 9, and 8 are activated, followed by a decrease in MMP, Bcl-2, and other MMPs, cIAP-1, and cIAP-2 levels well. Anthocyanins, which induce apoptosis, did not affect U937 cells with elevated Bcl-2 protein levels (Abaza et al., 2016). breast cancer (“MDA-MB-468″) cells treated with extracts (“*Vatica diospyroides”*) exhibit apoptosis as a result of Bax upregulation (Pal et al., 2010). An *Anemone raddeana* triterpenoid molecule, raddeanin A, is shown to activate apoptosis in stomach cancer cells. Raddeanin A upregulation pro-apoptotic protein (Bax) while decreasing the expressions of anti-apoptotic (BCL-xL) using molecular biology techniques. Additionally, in all three cell lines, this terpenoid increased the activity of caspase-3, -8, and -9, and the PARP enzyme (Orangi et al., 2016a). Non-small cell lung cancer cells” can be made to die by LA, which inhibits Bcl-2, FLIP, and XIAP while increasing “Bid” and caspase-3, -,8-9 (Chiu et al., 2015). LA is derived from the leaves of the Pinus koraiensis tree. The sBax and protein caspases up-regulated treated with Momordica cochinchinensis fruit extract, inducing apoptosis in MCF-7 (Chou et al., 2018). Using 34 HL-60 cells, ethyl acetate extract from Uncaria tomentoa (ethyl acetate (EA) extract) was found to activate the intrinsic pathway by compressing Matrix Metallo Protein and lowering anti-apoptotic (Bcl-XL protein), as well as by raising the membrane-bound Fas and activating caspase-8 (Sui et al., 2015). Protoberberine activated pro-apoptotic caspases, promoting cell death (apoptosis) in tongue cancer cells. Increased ratio (Bax/Bcl-2) and altered matrix protein were also observed (Pan et al., 2005). Two breast cancer cells were injected into athymic nude mice, and wogonin was found to have an anti-cancer effect *in vivo*. After 4 weeks of treatment, the xenograft burden was reduced by 88 percent (Chung et al., 2017a). Apoptosis in “breast cancer” (MCF-7) cells is caused by the methanolic fractions of *Scrophularia oxystepala* (Alshammari et al., 2020a). oleandrin, a carcinogenic glycoside, kills cancer cells in the U2OS and SaOS-2 lines by inducing cell apoptosis through the ROS Generation as well as the damage of Mitochondrial membrane potential, which discharges hydrolytic Enzymes into the cytosol and regulates the proteins (decreases the Bcl-2 level and increase Bax) along with caspases (caspases 9 and 8 and -3 (Liu et al., 2004).

**TABLE 1:** Plant extracts/fractions as apoptotic inducers for breast cancer (*in vitro*). updated and improved from (Israels and Israels, 2000).

Plant name	Extract/Fraction	Part used	Growth inhibition conc (μg/ml)	Target cell lines	Mechanism of cell death	References
Allium atroviolaceum	Methanolic Extract	Flower	500 μg/ml (70% GI)	MCF-7, MDA-MB-231	- Induces apoptosis	Ali et al. (2012)
					- Modulating Cell Cycle Arrest	
					- Caspase-Dependent and p53-Independent Pathway	
Phaseolus vulgaris (black turtle bean)	Extract	Seeds	50 μg/ml	MCF-7 and MDA-MB231	- Upregulation of Bax and downregulation of Bcl-2 and Bcl-xL	Mou et al. (2011)
					- Activation of caspase -3/7	
Ganoderma lucidum	Ethanol extract	Chipped fruiting bodies	234 μg/ml	MCF-7	- Induces cell cycle arrest and apoptosis -Up‐regulation of p21/Waf1 and down‐regulation of cyclin D1	Huang et al. (2008)
					- Up‐regulation of pro‐apoptotic Bax protein	
Echinophora Platyloba	Methanol Extract	Leaves	25 μg/ml	MDA-MB-231	- Induces Apoptosis and Cell Cycle Arrest at S-Phase	Bagheri et al. (2018)
					- Up-regulation of bax and p27	
					- Down-regulation of bcl-2	
Morinda Citrifolia	Ethyl-acetate extract	Fruit	25 μg/ml	MCF-7, MDA-MB-231	- Arrested the cell cycle in the G1/S phase in MCF-7 and G0/G1 phase in MDA-MB-231 cells	Kim et al. (2009)
					- Downregulation of intracellular “ROS” and “mitochondrial membrane potential”	
Fragaria ananassa Strawberry	Methanolic extract	Fruit	NA	T-47D	- Cleavage of MCL-1	Lee et al. (2009)
					- downregulation of BCL-xL	
					- Upregulation of expression of proapoptotic proteins such as BAX and BID	
					- Upregulation of p73	
					- Activation of Caspase 3 and Caspase 9	
Vatica diospyroides	Acetone and methanolic extracts	Fruit	1.60–17.45 μg/ml	MDA-MB-468	- Induces “apoptosis”	Azzopardi et al. (2017a)
					- “Up-regulation of Bax”	
Averrhoa Bilimbi	Methanolic extract	Fruit, Leaves	NA	MCF-7	- Anticancer Activity	Xue et al. (2013)
Carica papaya L	Aqueous Extract	Leaves	1319.25 μg/ml	MCF-7	- “Anti-proliferation” and “Apoptosis” Induction	Ahn et al. (2018)
Mimosa caesalpiniifolia	Ethanolic extract	Leaf	5.0 μg/ml	MCF-7	- Induces apoptosis	Somasagara et al. (2012)
					- “DNA fragmentation”	
Annona muricata	Aqueous extract	Leaves	NA	MCF-7, MDA-MB-231	- Induces apoptosis	Cheng et al. (2007)
Acanthopanax sessiliflorus	Hexane fraction	Stem bark	53.46 μg/ml 58.40 μg/ml	MDA-MB-231 and MCF-7	- “Non-apoptotic cell death” via mitochondria associated with both ROS dependent and independent pathways	Ho et al. (2009)
Phaleria macrocarpa	Ethyl acetate fraction	Fruit	18.10 μg/ml	MDA-MB-231	- Induce G0/G1 and G2/M cell cycle arrest	Chung et al. (2008)
					- Activation of caspase -8,9 and 3	
					- Upregulation of Bax, Bid	
					- cytochrome c, p21, p27, p53 and SMAC	
					- Downregulation of Bcl-2, Bcl-w, XIAP and survivin	
Stryphnodendron adstringens	Aqueous extract fraction	Leaves	76.31 μg/ml 186.83 μg/ml	MCF-7, MDA-MB-435	- Upregulation of Bax, caspase-9, active caspase-3, caspase-8, LC-3, and beclin-1	Hajiaghaalipour et al. (2015)
					- Downregulation of Bcl-2	
Avicennia Marina	Crude methanol extract and fraction	Leaves	250 μg/ml	MDA-MB 231	- DNA fragmentation.	Ma et al. (2016)
					- Decreased mRNA expression level of Bcl-2 and increased p53	
Salvia chloroleuca	Hexane and methylene chloride fractions	Roots	25.49–60.25 μg/ml	MCF-7	- Induced a sub-G1 peak	Wang et al. (2008)
					- DNA fragmentation	
					- ROS-mediated pathway	
Scrophularia oxysepala	Methanolic subfractions	Aerial parts	52.9–61.2 μg/ml	MCF-7	- Activation of caspase-3	Xu et al. (2019)
					- Downregulation of Bcl-2	
Artocarpus altilis	Diethyl ether extract	Wood	6.19 μg/ml	T-47D	- Induced apoptosis and sub-G1 phase formation	Ma et al. (2012)
Piper crocatum	Methanol extract	Leaves	44.25 μg/ml	T-47D	- Inhibition of p44/p42 phosphorylation	Tang et al. (2013)
Pistacia atlanticasub kurdica	Methanol	Fruits skin	1 mg/ml	T-47D	1. Activation of caspase 3	Zhao et al. (2017)
					2. Poly ADP ribose polymerase (PARP) cleavage	
Vitex rotundifolia	Extract/fraction	leave	6.30–63.09 μg/ml	MCF-7	1. Extrinsic and intrinsic pathway both activated	Khazaei et al. (2017b)
Vitex rotundifolia	fraction	leave	10-79.43 μg/ml	T47D	2. Extrinsic and intrinsic pathway both activated	Kumar et al. (2017)
Vitex negundo	Aqueous and Ethanolic extract	Leaves	200–300 μg/ml	MCF-7	3. Induced apoptosis	Hu et al. (2002)
Jatropha curcas	Ethanol extract	Root bark	36.55 μg/ml	MCF-7	4. Inducing anoikis	Birjandiana et al. (2018)
Vernonia amygdalina	Ethanol extract	Leaves	46–56 μg/ml	MCF-7 and MDA-MB-231	5. Induced apoptosis	Sharma et al. (2016)
					6. G1/S phase cell cycle arrest	
					7. Caspase-dependent	
Strobilanthes crispa	Hexane extract	Stem	42.5 μg/ml	MDA-MB-231	1. Induced apoptosis	Ranganatha et al. (2012)
Ixeris dentata	Methanol extract	-	100–200 μg/ml	T-47D, MCF-7, SK-BR-3, and MDA-MB-231	- Induced apoptosis	Azzopardi et al. (2017b)
					- *Via* Akt-NF-κB signaling	
Tinospora cordifolia	Chloroform fraction	Stems	28.09–35.06 μg/ml	MCF-7 and MDA-MB-231	1. ROS mediated apoptosis	Nair et al. (2016)
Smilax china	Ethanol extract	Bark	NA	MDA-MB-231	2. Suppression of metastasis	Zuhrotun et al. (2017)
					3. “Modulation of uPA, uPAR and TIMP expression”	
Bauhinia ungulata	Different fractions	Stem	23.47 µg/mL	4T1	4. Anti-tumor	Silva et al. (2014)
					5. Antimetastatic	
					6. Decreasing the MMP-2 activity	
157Nicotiana glauca	Dichloromethane fraction	Stem	17.98 μg/ml	MCF-7	- Anti-Metastatic	Kim et al. (2018)
Euphorbia humifusa	Ethyl acetate fraction	Whole plant	5 μg/ml	MDA-MB-231	7. Inhibition of NF-κB activity	Thamizhiniyan et al. (2015)
					8. Induced matrix metalloproteinase (MMP)-9 mRNA expression	
Withania coagulans	Ethyl acetate	Aerial with fruit	NA	MCF-7, MDA-MB-231	- Inhibited TNF-α induced NFκB activity	Kavitha et al. (2017)
Astragalus membranaceus	Water- ethanol extract	Roots	NA <100 μg/ml	MCF-7, SK-BR-3, and MDA-MB-231	- Anti-proliferative	Sabino et al. (2018)
					- Induced apoptosis	
					- Inhibition of PI3K/AKT/mTOR signaling pathway	
Dillenia suffruticosa	Ethyl acetate extract	Roots	36 μg/ml	MCF-7	- “Induces apoptosis via inhibition of AKT and ERK”, and activation of JNK	Momtazi-borojeni et al. (2013)
Catharanthus roseus	Methanol extract	Leaves	> 200 μg/ml.	MDA-MB-231	- Anti-invasive	Tayarani-Najarana et al. (2013)
					- Suppressed the MMP-2 and MMP-9 activity	
Forsythia koreana	Methanol extract	Fruit and leaves	NA	MDA-MB-231	- “Suppressed invasion and MMPs activities”	Orangi et al. (2016b)
					- Inhibited the receptor activator of nuclear factor kappa-B	
Origanum majorana	Ethanolic extract	Leaves	NA	MDA-MB-231	- “Anti-invasive and anti-metastatic	Arung et al. (2009)
					- Downregulates the phosphorylation of IκB, nuclear level of NFκB and Nitric Oxide (NO) production”	
Brassica oleracea	Extract	-	NA	MDA-MB-231	- Anti-invasive	Wicaksono et al. (2009)
					- Suppressed TPA-induced MMP-9 activity	
Salvia triloba	Ethanolic crude extracts	Whole plant	NA	MCF 7	- Antiangiogenesis	Rezaei et al. (2011)
					- Inhibited the expression of VEGF at the mRNA and protein level	
Eugenia jambolana	Ethyl acetate fractions	Seeds	25 μg/ml	MCF-7, MDA-MB-231	- Suppression of VEGF-induced angiogenesis	Chaudhry et al. (2019a)
Musa paradisiaca	Ethyl acetate fractions	Roots	60 μg/ml	MCF-7,MDA-MB-231	- Suppression of VEGF-induced angiogenesis	Chaudhry et al. (2019a)
Buxus sempervirens	Acetonic extract	Leaves and flowers	7.74–12.5 μg/ml	MCF7, T47D, MCF10CA1a, and BT-20	- Induces apoptosis	Chaudhry et al. (2019b)
					- Cell cycle arrest, Autophagy	
Cucurbita ficifolia	Chloroform	Fruit	90 μg/ml	MCF-7	- FADD; BAK; BAX; caspase-3, -9, and -8 Increased	Arulvasu et al. (2010)
Cyperus rotundus L.	Ethanol	Rhizome	200 μg/ml	MDA-MB-231	- Upregulation: Bax; DR5; activation of Bid; activation of caspase-3, -9, and -8 downregulation: Bcl-2; survivin; MMP	Engel et al. (2014)
Euphorbia hirta L.	Methanol	Whole plant	25.26 μg/ml	MCF-7	- Activation of caspase-2, -6, -8, -9, and -3 Increased	Wong et al. (2013)
Oldenlandia diffusa	Methanol and butanol	Whole plant	0–20 μg/ml	MCF-7	- Bax; activation of caspase-8 and -7 Increased and down regulation Bcl-2	Koh et al. (2017)

## 6 Marine extracts/fractions as apoptosis regulator

Aquatic environments, especially marine species, contain abundant various natural compounds which may have therapeutic significance. However, recent marine biomaterials experience demonstrates that ocean life seems to be an untapped resource. Bioactive compounds found naturally are the most effective. Apart from a few exceptions, most compounds used in medicines originate through biological and chemical modifications rather than from the initial natural substance. Our previous studies confirm apoptosis induction in the cancer cell (*in-vitro*) by marine extracts and fractions. The Aaptos sp., marine (fraction) causes the DNA fragmentation in the MCF-7 cell line, which is a remarkable feature of apoptosis (late). Similarly, marine sponges (whole) extracts and fractions also induce the fragmentation in DNA in breast cancer (MCF-7) cell lines. The details information mention in Table 2. The “*Stichopus chloronotus”* and “*Holothuria nobilis”* fractions induced cytotoxicity via apoptosis induction in cervical cancer (HeLa) cells shows the remarkable potential of marine phytochemicals in the induction of apoptosis. The “*Bruguiera gymnorrhiza”* extracts potential phytochemicals that trigger DNA fragmentation, leading to the induction of apoptosis in MCF-7 cells. The other marine species, “*Acanthaster planci”* sp*.,* and “*Diadema setosum”* sp., fractions Induction of apoptosis in HeLa cells. The “*Xylocarpus moluccensis”* induce the triggering of the Extrinsic pathway of apoptosis which causes the primary factor for cytotoxicity of fractions in human hepatocellular carcinoma (HepG2) and human cervical cancer cell line (HeLa). Some alkaloid chemicals discovered in traditional medicines, tetrandrine (TET) and cepharanthine (CEP), operate as apoptotic agents. There is reduction in the progression of malignant tumors. When viewing human leukemia cells treated with alkaloids, Xu et al. found that both initiator and effector caspases were dramatically increased (caspase-3 and& -6). Saikosaponin A, a triterpenoid saponin, was studied by Kim and colleagues to see if it could kill human colon cancer cell lines. According to the researchers’ findings, the activation of Bax and Bid proteins and the activation of caspase-2, -8, and -9 in saikosaponin A increased apoptosis in cells. Emodin (40 mg/kg/day) suppressed tumor weight in “nude mice xenografts” carrying “LS1034” colon cancer cells *in vivo*, according to *in vitro* findings. “Anti-apoptotic” proteins “Bcl-2” and Bcl-xL, Bax, XIAP and cFLIP are reduced in “HT-29”, “HCT-116” and “SW480” cells by the flavonoid compound Casticin. The “OVCAR-3” and “SKOV-3” (ovarian cancer) cell lines were exposed to kaempferol (kaempferol), a flavonoid, the levels of apoptosis-inducing factors like proteins (Bax, caspase-3, -8, & -9) were significantly increased. In contrast, apoptosis inhibiting proteins decreased in these cells. Similar outcomes were found when combined with the anti-inflammatory agent TRAIL.

**TABLE 2:** Marine (sponges) extracts/fractions as apoptotic inducers for various cancer cell lines (*in vitro*). Improved and updated from (Shin et al., 2017) (Calcabrini et al., 2017).

Marine species	Cell line	Conc. Range IC_50_ (μM)/μg/mL)	Phosphatidylserine externalization (PS) (initial apoptosis)	DNA fragmentation (late Apoptosis)	Caspase activation	PARP cleavage	References
*Aaptos sp., fractions*	MCF-7	5.0–25.00 μg/ml	√	√			Ansari et al. (2017)
*Stryphuous ponderosus AaptosTheonella Xestospongia*	MCF-7	8.49 μg/ml	√	√			Nho et al. (2015)
		7.61 μg/ml					
		21.88 μg/ml					
		97.72 μg/ml					
*Stichopus chloronotus*	HeLa	6.71–8.54 μg/ml	√	√			Santos et al. (2017)
*Holothuria nobilis*	HeLa	14.76–2.54 μg/ml	√	√			Santos et al. (2017)
*Bruguiera gymnorrhiza*	MCF-7	3.39–37.15 μg/ml	√	√			Yasser et al. (2015)
*Acanthaster planci*	HeLa	0–18.93 μg/ml	√				Shin et al. (2016)
*Diadema setosum*	HeLa	0–30 μg/ml	√				Shin et al. (2016)
*Xylocarpus moluccensis*	HeLa, HepG2	0.22–74.13 μg/ml	√				Ihsan-ul- HaqMirza et al. (2013)
*Aaptos* sp.	THP-1	50–200 μM	√				Zhou et al. (2018)
*Aaptos* sp.	THP-1	10–25 μM	√				Zhou et al. (2018)
*Aaptos* sp.	THP-1	10–25 μM	√				Zhou et al. (2018)
*Amphibleptula* sp.	AsPC-1	2.4	μM	√	3 and 7		Tor et al. (2015)
	BxPC-3	2.4	μM	√	3 and 7		
	PANC-1	2.4 μM		√	3 and 7		
*Aplysina aerophoba*	Raji	50		√	3 and 7	√	Eltayeb et al., (2016)
	U937	50 μM		√		√	
*Australian spongia* sp*.*	AsPC-1	6.8 μM		√	3 and 7		Kim et al. (2016)
*Australian spongia* sp*.*	PANC-1	6.8 μM		√	3 and 7		Kim et al. (2016)
*Australian spongia* sp.	MIA PaCa-2	6.8 μM					Kim et al. (2016)
*Australian spongia* sp.	BxPC-3	6.8 μM					Kim et al. (2016)
*Cacospongia mycofijiensis*	MDA-MB-435	0.1 μM			3		Dhaheri et al. (2013)
*Cacospongia scalaris*	HeLa	10 μg/ml			3		Rose et al. (2005)
	T47D						
*Candidaspongia* sp*.*	U251 HCT116	0.05–0.10 μM	√		3 and 12		Zihlif et al. (2013)
*Callyspongia* sp.	HL-60	31.0–77.5 μM		√			Harsha et al. (2017)
*Crambe crambe*	HepG2	0.5–2.5 μM	√		3		Ait-Mohamed et al. (2011)
*Crambe crambe*	HepG2	0.5–2.5 μM	√		3		Ait-Mohamed et al. (2011)
*Crambe crambe*	HepG2	0.5–2.5 μM	√		3		Ait-Mohamed et al. (2011)
*Dactylospongia elegans*	U937 HL-60	5–15 μM		√			Alshammari et al. (2020b)
*Dinophysis fortii and*	Hep3B	0.01 μg/ml	√		3, 8, and 9		Park et al. (2014)
*Dinophysis acuminata*	U937	0.008–0.010 μg/mL	√	√	3	√	Kwan et al. (2016), Chung et al. (2017b), Kim et al. (2008), Moon et al. (2008)
*Fascaplysinopsis* sp*.*	K562 normoxic and hypoxic conditions	0.01–0.2 μM	√		3 and 9		Hudayah et al. (2017), Chaudhry et al. (2018)
*Fasciospongia cavernosa*	Hela	10 μg/ml	√		3		Hudayah et al. (2017)
	T47D	10 μg/ml					
*Forcepia* sp.	CA46, Ramos, Daudi, HL-60, MDA-MD-231, MCF-7, HCT-116, HT-29	0.1 μM		√			Chaudhry et al. (2020b)
*Geodia japonica*	HL-60	1.6–25 μg/ml	√	√	3		Chaudhry et al. (2020c), Chaudhry et al. (2021b)
	HT-29	5–30 μM		√			
*Geodia japonica*	HL-60	4 μg/ml			3		Chaudhry et al. (2021c)
*Hippospongia metachromia*	HCT116	2.5–10 μM	√	√	3 and 8		Dyshlovoy et al. (2014), Calcabrini et al. (2017)
	PC-3	2–10 μM		√			Guzman et al. (2015)
*Spirastrella spinispirulifera*	MCF-7	0.0002–0.0005 μM		√	2,5,7,8, 9		Florean et al. (2016)
*Hyrtios erecta*	Jurkat	0.0002 μM		√	2, 3, 7, 8 ,and 9		Guzman et al. (2013)
	L3.6pL	0.00001–0.01 μM		√			Mooberry et al. (1999)
*Hyrtios sp.*	K562	1.4–5.6 μM		√	3, 8, and 9		
	DU145	0.01–1 μg/ml		√	3, 8, and 9		De Stefano et al. (2012)
	PC-3	0.01–1 μg/ml		√	3, 8, and 9		Trisciuoglio et al. (2008)
	LNCaP	0.01 μg/ml		√			
	T24	0.1–0.8 μg/ml	√	√	3 and 9		Umeyama et al. (2010)
	A498	0.5–3 μM		√	3, 8 and 9		Roel et al. (2016)
*lanthella* sp*.*	HUVEC	0.01–1 μM		√	3 and 7		Kong et al. (2008)
*Ircinia ramose, Psammocinia* sp.	Jurkat	0.01 μM		√	3, 8 and 9		Shin et al. (2008)
*Jaspis* sp.	Bel-7402	0.5 μM		√			Kim et al. (2008)
	HepG2	10–20 μM					
*Jaspis stellifera*	K562	0.012–0.054 μM	√		3 and 9	√	Moon et al. (2008), Ben-Califa et al. (2012), Del Poggetto et al. (2015)
	A549	0.02–1 μM	√		3 and 7	√	
	SF295	0.04–1 μM	√			√	
*Jaspis sp. Pachastrissa* sp.	B16	5 μM	√	√	3 and 9	√	Cheung et al. (2010), Zhang et al. (2012)
	HaCaT	5 μg/ml			3		
*Lanthella* sp.	HL-60	19.9 μM		√			Liu et al. (2005)
*Lanthella* sp*.*	HL-60	21.3 μM		√			Liu et al. (2005)
*Lanthella* sp*.*	HL-60	21.5 μM		√			Liu et al. (2005)
*Leiodermatium* sp*.*	AsPC-1	0.01 μM		√			Liu et al. (2006)
	BxPC-3	0.01 μM		√	3		
	MIA PaCa-2			√	3		
*Leucetta chagosensis*			√		3, 8 and 9		Do et al. (2014)
*Monanchora pulchra*	HeLa	1.39–2.01 μM		√	3 and 7		Lee et al. (2015)
*Mycale* sp*.*	32D	0.1 μM					Lu et al. (2007)
*Negombata magnifica*	MKN45 NUGC-4	− 10		√	3 and 7		Schneiders et al. (2009)
		0.01–10 μM		√			
*Oceanapia sagittaria*	MCF-7	0.5–2.5 μM		√			Schyschka et al. (2008)
*Petrosia sp.*	SK-MEL-2	0.1–0.3 μg/ml		√	3 and 9		Rothmeier et al. (2010)
*Psammaplysilla* sp*.*	Human endometrial Ishikawa			√			Schumacher et al. (2010)
*Psammaplysilla*	Ishikawa	1 μM				√	Schumacher et al. (2010)
	ECC1	1 μM					
*Pseudoceratina* sp*.*	K562	7.7–30.8 μM		√	3 and 9		Wu et al. (2016)
*Rhabdastrella globostellata*	HUVEC	1–10 μM			3 and 7		Huang et al. (2016)
*Rhabdastrellav globostellata*	HL-60		√		3	√	Wu et al. (2015)
	HL-60	10–25 μM	√		3, 8 and 9		Aoki et al. (2006)
*Rhizochalina incrustata*	HT-29	1–6 μM	√		3		Chinen et al. (2010)
	THP-1	1–10 μM	√				Wei et al. (2008)
	PC-3	0.5–4 μM	√		8		
	DU-145	0.5–4 μM	√		8		
	22Rv1	0.5–4 μM	√		8		Chen et al. (2017)
	VCaP	0.5–4 μM	√		8		
*Sarcotragus*	SK-MEL-2	25–50 μM		√	3 and 9	√	Wang et al. (2016)
*Siphonochalina* sp*.*	HepG2	17.18 μM		√	3		Salma et al. (2009), Tang et al., (2014)
	HCT-116	14.8 μM		√	3		
*Siphonochalina* sp.	HepG2	24 μM		√	3		Salma et al. (2009)
	HCT-116	19.8 μM μM		√	3		
*Smenospongia aurea*	Calu-1	0.05–0.1 μM	√	√			Yoo et al. (2012)
*Stelletta* sp.	U937	17.2–103.3 μM	√	√	3, 8 and 9		Nguyen et al. (2009)
*Xestospongia* sp.	H460	5–40 μM		√	3	√	LaBarbera et al. (2009), Guzman et al. (2016)
	U373MG	0.0031 μM					

## 7 Marine isolated targeted compounds as apoptotic inducers (FDA approved/treatment phase)

Besides extracts/fractions, the various marine-based drug also show apoptosis-inducing potential. And over 50 years since cytarabine was authorized, four marine-derived drugs have been approved (Hu et al., 2013). Some other items have been included in the marine anticancer medicinal route in clinical studies in different phases of development as we focused on apoptotic inducers. Table 3 shows the list of respective drugs (approved or in clinical trials) having the potential for inducing apoptosis. These include; Brentuximab2 vedotin (AdcetrisTM), Brentuximab2 vedotin (AdcetrisTM), as “Antibody-drug3 conjugate in a mixture of anti-CD30 antibody and monomethyl auristatin E (MMAE), isolated from sea hare Dollabella auricularia/cyanobacteria” used for Blockage in cell cycle progression (G2 to M) induce induction of apoptosis phase III and I treatment of classical HL in a mixture using chemotherapy for curing HL and chronic large cell anaplastic lymphoma. Enfortumab vedotin, Marizomib, Polatuzumab Vedotin, use in phase III. Besides, GSK2857916, Aplidine, plitidepsin (Aplidin), Ladiratuzumab vedotin, Glembatumumab vedotin, Denintuzumab mafodotin, in phase II trials. However, Pinatuzumab vedotin, ASG-15ME, CEP-2563, Lifastuzumab vedotin, and Vandortuzumab vedotin were discontinued after phase I used for lymphoma, bladder, ovarian, as Peritoneal cancer. Vandortuzumab isolated from Cyanobacterium. (Caldora penicillata) acts as apoptotic inducer and mitotic inhibitor for Prostate cancer.

**TABLE 3:** Marine base drugs (FDA Approved/treatment Phase) as Apoptotic inducers.

Drug	Source (marine)	Mechanism of action	Progress status	Treatment for	Ref
Brentuximab2 vedotin (AdcetrisTM)	“Antibody-drug3 conjugate in a mixture of anti-CD30 antibody and monomethyl auristatin E (MMAE), obtained from sea hare Dollabella auricularia/cyanobacteria”	Blockage in cell cycle progression (G2 to M) induce “induction of apoptosis”	Approved by FDA (United States 2018); Also, phase III and I treatment of classical HL in a mixture using chemotherapy	“It is utilized for curing HL and chronic large cell anaplastic lymphoma”	Dyshlovoy et al. (2016)
Enfortumab vedotin	Cyanobacterium Caldora penicillata	By interaction with the microtubular network Arrest the cell cycle, and eventually apoptosis	Phase III	For Urothelial cancers	Dyshlovoy et al. (2016)
Marizomib	Actinomycete	“Act as apoptosis	Phase III	Used for “multiple	Dyshlovoy et al. (2016)
	Salinispora tropica	Stimulants” and		myeloma and	
		“inhibits proteasome”		Glioblastoma”	
Polatuzumab	Cyanobacterium	As a apoptosis stimulants	Phase III	For the treatment	Dyshlovoy et al. (2016)
vedotin	Caldora penicillata	arrest mitosis		of diffuse large B cell	
		also tubulin		lymphoma	
		inhibition micro-tubulin			
		Polymerization			
GSK2857916	Cyanobacterium	Apoptosis stimulant via	Phase II	Multiple myeloma	Hood et al. (2001)
	Caldora penicillata	“mitosis inhibitors tubulin			
		polymerization inhibitors”			
Aplidine	Tunicate Aplidium	“Apoptosis stimulants”, cell	Phase II	“Multiple myeloma	Hood et al. (2001)
plitidepsin	alpicans	cycle inhibitors protein		precursor cell	
(Aplidin)		“synthesis inhibitors”		Lymphoblastic”	
		“vascular endothelial		leukemia-lymphoma	
		growth factor receptor-1			
		Antagonists”			
Ladiratuzumab	Cyanobacterium	Apoptosis stimulants	Phase II	For Breast cancer	Hood et al. (2001)
vedotin	Caldora penicillata	“mitosis inhibitors”			
Glembatumumab	Cyanobacterium	Apoptosis stimulants	Phase-II	Brain cancer and Breast	Hood et al. (2001)
vedotin	Caldora penicillata	mitosis inhibitors tubulin		osteosarcoma	
Denintuzumab	Cyanobacterium	Act as “Immunomodulators”		uveal melanoma	
mafodotin	Caldora penicillata	mitosis inhibitors also inhibitors of tubulin polymerization	Phase II	Diffuse large B-cell	Konishi et al. (2009); Dyshlovoy et al. (2016)
			(discontinued)	lymphoma	
Pinatuzumab	Cyanobacterium	Apoptosis stimulants	Phase I	Chronic lymphocytic	Hood et al. (2001); Dyshlovoy et al. (2016)
vedotin	Caldora penicillata	mitosis inhibitors tubulin	(discontinued)	leu emia diffuse large	
		inhibitors tubulin		B cell lymphoma nHL	
		polymerization inhibitors			
ASG-15ME	Cyanobacterium	Apoptosis stimulants	Phase I	Bladder cancer	Kijjoa et al. (2007); Dyshlovoy et al. (2016)
	Caldora penicillata	mitosis inhibitors tubulin	(discontinued)	urogenital cancer	
		inhibitors tubulin			
		polymerization inhibitors			
CEP-2563		Protein tyrosine kinase	Phase I	Solid tumors	Kijjoa et al. (2007); Dyshlovoy et al. (2016)
		inhibitors	(discontinued)		
Lifastuzumab	Cyanobacterium	Apoptosis stimulants	Phase I	For various cancer Fallopian tube cancer	Dyshlovoy et al. (2016)
vedotin	Caldora penicillata	mitosis inhibitors tubulin	(discontinued)	non-small cell lung	
		inhibitors tubulin		Cancer, ovarian cancer	
		polymerization		Peritoneal cancer	
Vandortuzumab	Cyanobacterium	Apoptosis stimulants	Phase I	Prostate cancer	Dyshlovoy et al. (2016)
vedotin	Caldora penicillata	mitosis inhibitors	(discontinued)		
		polymerization inhibitors			

## 8 Conclusion

The potential use of natural substances as either a combination treatment with standard therapies or as an individual derivative molecule as anticancer medicine requires an understanding of cancer initiation and activation of cancer development, the role of signalling pathway in activation of cancer initiation and progression, pro-oncogenes, and oncogenes. Since apoptosis is a crucial regulating mechanism for normal cells, any disruption in this process might lead to unchecked cell growth. An in-depth mechanistic knowledge of apoptotic signalling pathways is necessary for the creation of efficient cancer therapies. Plant extracts, marine extracts/fractions, and the chemicals described in this research may represent a promising and efficient approach to cancer treatment. Researchers found that natural product derivatives may have an important role in the prevention and treatment of several different types of cancer. To verify their overall usefulness as potent chemotherapeutic drugs, however, more preclinical and clinical research are required. It is interesting to note that natural compounds produced from plant extracts/marine can be used in combination with possible anticancer treatment to enhance drug sensitivity in aggressive and resistant kind of cancer. Due to the promising results seen when using natural goods in conjunction with cancer medications, intensive study into the therapeutic uses of natural products is required to get the best possible outcomes in individualized, targeted cancer care. Similarly, the use of more than one compounds as multitargeted signaling pathway inhibitors or activator should be consider with one signaling target as apoptosis pathway. However, natural chemical derivatives that induce apoptosis, along with cutting-edge drug delivery methods like polymeric nanoparticles, may yield impressive results. Compounds with low solubility and bioavailability are less likely to be effective in the development of chemotherapy drugs. In conclusion, this study aims to bring to the notice of scientists and researchers the numerous beneficial impacts of natural products in the development of novel and safe pharmaceuticals or in combine delivery technique with anticancer treatments for probable sensitization of cancer therapy. It might also serve as a solid basis for further research into natural compounds in cancer therapeutics.
